# The fecal microbiota of piglets during weaning transition and its association with piglet growth across various farm environments

**DOI:** 10.1371/journal.pone.0250655

**Published:** 2021-04-27

**Authors:** Diana Luise, Mathilde Le Sciellour, Arnaud Buchet, Rémi Resmond, Charlène Clement, Marie-Noelle Rossignol, Deborah Jardet, Olivier Zemb, Catherine Belloc, Elodie Merlot

**Affiliations:** 1 Department of Agricultural and Food Sciences (DISTAL), Agricultural, Environmental, Food Science and Technology, Alma Mater Studiorum, University of Bologna, Bologna, Italy; 2 PEGASE, INRAE, Institut Agro, Saint Gilles, France; 3 Cooperl Arc Atlantique, Lamballe, France; 4 @BRIDGE, GABI, INRAE, Jouy-en-Josas, France; 5 GenPhySE, INRAE, Castanet Tolosan, France; 6 BIOEPAR, INRAE, ONIRIS, Nantes, France; Wageningen Universiteit, NETHERLANDS

## Abstract

This study describes the fecal microbiota from piglets reared in different living environments during the weaning transition, and presents the characteristics of microbiota associated with good growth of piglets after weaning. Fecal samples were collected pre- (d26) and post-weaning (d35) from 288 male piglets in 16 conventional indoor commercial farms located in the West of France. The changes one week after weaning on the most abundant microbial families was roughly the same in all farms: alpha diversity increased, the relative abundance of *Bacteroidaceae* (-61%), *Christensenellaceae* (-35%), *Enterobacteriaceae* (-42%), and *Clostridiaceae* (-32%) decreased, while the relative abundance of *Prevotellaceae* (+143%) and *Lachnospiraceae* (+21%) increased. Among all the collected samples, four enterotypes that were ubiquitous in all farms were identified. They could be discriminated by their respective relative abundances of *Prevotella*, *Faecalibacterium*, *Roseburia*, and *Lachnospira*, and likely corresponded to a gradual maturational shift from pre- to post-weaning microbiota. The rearing environment influenced the frequency of enterotypes, as well as the *relative* abundance of 6 families at d26 (including *Christensenellaceae and Lactobacillaceae*), and of 21 families at d35. In all farms, piglets showing the highest relative growth rate during the first three weeks after weaning, which were characterized as more robust, had a higher relative abundance of *Bacteroidetes*, a lower relative abundance of *Proteobacteria*, and showed a greater increase in *Prevotella*, *Coprococcus*, and *Lachnospira* in the post-weaning period. This study revealed the presence of ubiquitous enterotypes among the farms of this study, reflecting maturational stages of microbiota from a young suckling to an older cereal-eating profile. Despite significant variation in the microbial profile between farms, piglets whose growth after weaning was less disrupted were, those who had reached the more mature phenotype characterized by *Prevotella* the fastest.

## Introduction

In pig husbandry, weaning is a critical health-challenging period for piglets, combining nutritional, environmental, and social changes. Intestinal homeostasis plays a major role in maintaining of general health and preventing infectious diseases [1], and this is particularly relevant for piglets at weaning, for which the abrupt environmental changes may generate a dysbiosis, favorable to pathogenic bacteria, thus causing post-weaning diarrhea (PWD) [2]. Even in the absence of PWD, weaning is associated with a transient reduction in growth due to temporary malabsorption of nutrients, often combined with a subclinical deterioration in animal health. Robust pigs are those whose growth and health are little affected by weaning or display good resilience (i.e. the ability to recover rapidly). The post-weaning live weight trajectory appears to be a pertinent indicator of piglet robustness and is associated with the diarrhea score of weaned piglets [3].

The role of gut microbiota in moderating host growth and health is now well recognized. In pigs, characteristics of gut microbiota are associated with differences in feed efficiency [4–6] and with average daily gain (ADG) observed among weaned piglets [7, 8]. Early developmental history in turn influences microbiota. For example, microbiota richness and the abundance of some genera differ between low and normal birth weight piglets until weaning age [9]. When it comes to animal health, increasing evidence suggests that disturbances to microbiota can result in a higher propensity to develop certain health disorders [2, 10]. In pigs, animals susceptible to PWD can be discriminated from healthy ones by their fecal microbiota as early as one week after birth [11]. However, it is not easy to clearly identify the microbiota characteristics associated with health, especially when considering subclinical situations, which are only revealed by growth impairment and not through the detection of specific pathogens [9]. This characterization is made difficult by the dependence of microbiota on the environment. In mice, the correlations observed between microbiome composition and health are highly variable across different facilities [12]. Environmental influences, resulting from biotic or abiotic differences as well as from variations in rearing practices among facilities, are unavoidable in pig farms. For example, the impact of the rearing environment on the microbiota of piglets was shown for the genera *Oscillospira*, *Megasphaera*, *Parabacteroides*, and *Corynebacterium*, whose abundances differed among pigs from different farms [13]. It is therefore important, when looking for observed associations between the microbiota and biological characteristics of the individuals that host it, to verify the stability of these associations from one farm to another [14].

Thus, this study aimed to determine whether the changes observed in microbiota after weaning are the same across various rearing environments, and to capture features of microbiota associated with the robustness of piglets after weaning that are consistently observable across these different farms. For this purpose, data were collected from a high number of animals (288) raised in 16 conventional indoor commercial farms, all geographically located in the West of France (Table 1). Post-weaning growth performance was used as a proxy for piglet robustness.

**Table 1:** Characterization of the farms, practices, and treatments during the study, and growth of animals involved in the study.

	Number of sows (and batches)	Age at creep feed start (days)	Allotment of experimental pigs post-weaning	Piglet collective antimicrobial treatment ^1^	Days with reported diarrheas^2^	Weaning weight (kg)^3^	d26-d48 ADG (g/d)^3^	d26-d48 relative ADG (g/kg/d) ^3^
Farm 1	287 (7)	7 (+ peat)	1 pen of 70–90 pigs	Linco + Spect (f)	4	8.2 ± 0.4	265 ± 17	32 ± 2
Farm 2	158 (7)	8	6 pens of 11–16 pigs	-	2	6.7 ± 0.4	208 ± 20	30 ± 3
Farm 3	356 (7)	(peat)	1 pen of 40 pigs	-	3	7.4 ± 0.4	285 ± 17	38 ± 2
Farm 4	222 (7)	10	1 pen of 30 pigs	Col + Doxy (f)	1	8.6 ± 0.3	295 ± 29	35 ± 4
Farm 5	110 (7)	6	1 pen of 18 pigs	-	2	7.1 ± 0.4	295 ± 17	42 ± 2
Farm 6	187 (7)	birth	1 pen of 50 pigs	-	11	7.9 ± 0.4	221 ± 22	29 ± 3
Farm 7	177 (7)	10	1 pen of 50–60 pigs	-	4	8.5 ± 0.2	232 ± 22	27 ± 2
Farm 8	131 (7)	1	1 pen of 22 pigs	Col (w)	NA	8.8 ± 0.2	357 ± 15	40 ± 1
Farm 9	186 (7)	4	6 pens of 20 pigs	-	3	7.7 ± 0.3	316 ± 15	42 ± 2
Farm 10	131 (7)	8 (+ peat)	1 pen of 57 pigs	-	9	8.3 ± 0.3	315 ± 22	38 ± 3
Farm 11	171 (7)	3	1 pen of 28 pigs	-	NA	8.6 ± 0.4	337 ± 24	40 ± 2
Farm 12	106 (7)	4	3 pens of 30–40 pigs	Col + Amox (f)	4	8.0 ± 0.3	311 ± 11	40 ± 2
Farm 13	116 (3)	4	2 pens of 10–11 pigs	Tula (in)	1	8.3 ± 0.3	445 ± 28	53 ± 2
Farm 14	191 (7)	5	1 pen of 30 pigs	Col + Doxy (f)	1	8.7 ± 0.3	430 ± 24	49 ± 2
Farm 15	116 (4)	7 (+peat)	1 pen of 18 pigs	Apra + Sulfa + Trim (f)	0	9.1 ± 0.4	352 ± 25	39 ± 2
Farm 16	189 (7)	2	2 pens of 19–20 pigs	Col + Doxy (f)	6	9.1 ± 0.4	385 ± 18	43 ± 2

^1^ Antimicrobials administered collectively between d27 and d35 in feed (f), water (w), or by intramuscular injection (in). Amox: Amoxicillin, Apra: Apramycin, Col: Colistin, Doxy: Doxycycline, Lynco: Lincomycin, Trim: Triméthoprim, Tula: Tulathromycin, Spect: Spectinomycin, Sulfa: Sulfadiazine.

^2^ Number of days over the d27 to d48 period, during which farmers noticed pigs with digestive disorders in the post-weaning room.

^3^ d26-d48 ADG: Average daily gain calculated between 26 and 48 days of age, d26-d48 relative ADG: d26-d48 ADG / d26 weight. Values correspond to the mean ± the standard error of the mean of the animals recruited for the study (n = 18 per farm).

## Materials and methods

### Ethics approval

A competent ethics committee in animal experimentation has approved the experiment (authorization #CERVO-2016-6-V, committee of the national veterinary school of Nantes, France).

### Experimental design and farm characterization

A total of 288 Pietrain x (Large White x Landrace) piglets were included in the study. Piglets were sampled from January to June 2015 in 16 commercial farms located in the west of France (within a 90 km radius of the laboratory) and members of the Cooperl cooperative. Farms with a breeder-fattener structure, with more than 100 sows, that wean piglets at 4 weeks of age were chosen. The GTE database was used to select farms representing the diversity of performances for post-weaning average daily gain (ADG). Other characteristics of the farms were recorded but the heterogeneity in practices, feeding, pen size and design, vaccination scheme, and antimicrobial administration post-weaning was great and the farm pool was not big enough to make homogeneous groups. A summary of the information concerning farm characteristics and practices is reported in Table 1, and more detailed information, especially regarding feeding, is provided in the S1 Table. A questionnaire administered to the farmer, and a consultation of the farm veterinary record made it possible to more accurately describe the farmer’s practices and the sanitary status of the herd.

Overall, farms used different but conventional lactation diets for sows, and starter and weaner diets for piglets. Piglets received creep feed starting from 4 to 10 days of age, depending on the farm, and some farms distributed peat starting from 3 to 4 days of age. During the first week after weaning, three different feed formula were used in the farms (S1 Table): 3 farms used the “Premium” diet, 8 used the “Premium Acti” diet, and 5 the “Prem Dieto” diet. These 3 diets contained respectively 18.5, 18.5 and 17% proteins, 8, 7 and 6.5% fat, 2.8, 3.0 and 3.5% crude fibers, 5.5, 5.2 and 5.8% minerals, and 1.43, 1.43 and 1.40% lysine. All 3 diest had 11% humidity. Individual and collective medications administered to piglets were recorded during the study. None of the pigs received antimicrobial treatment until d26. During the first week after weaning, eight farms did not use any collective antimicrobial treatment (Table 1). In others, antimicrobials were administered during the first week post-weaning via an antimicrobial supplemented starter diet (6 farms), drinking water (1 farm), or on the day of weaning by intramuscular injection (1 farm, Table 1). This diversity of practices regarding antimicrobial use reflected the actual variability observed in French pig farms at that time [15]. This variability did not make possible the investigation of specific antimicrobials on fecal microbiota in this study.

In each farm, two days before weaning, 9 sows of different parities were chosen to reflect the demographic pyramid of the herd, and two apparently healthy and middleweight non-castrated males per litter were ear-tagged and included in the study (18 piglets per farm). At weaning (at 27 ± 2 days of age), piglets were transferred to the post-weaning rooms of their farms, where they were group-housed in groups of 10 to 80 pigs, depending on the farm, and the experimental piglets were always housed in the same post-weaning room (but sometimes in different pens, see Table 1).

### Animal data and sample collection

Piglets were individually weighed on days 26 (d26), 35 (d35), and 48 ± 2 days (d48). Individual feed intake could not be recorded because pigs were group-housed and farms were not equipped for such measurement. A fecal sample was individually collected on d26 and another on d35. Each ear-tagged piglet was caught individually, contained in the arms of one experimenter, and defecation was stimulated by introducing the tip of a thermometer into the rectum. The sample was collected in a sterile tube, kept on ice until arrival at the laboratory, and then stored at -80°C until analysis. For practical reasons, feces samplings were missing for one farm at d26 and one at d35. Piglet health status and diarrhea occurrence (presence of dirty pigs in the pen) was checked and recorded daily by farmers.

In this study, we used the post-weaning growth performance corrected from the pre-weaning growth trajectory (rADG) as a proxy for robustness to the weaning event. The relative average daily gain (rADG) from d26 to d48 was individually calculated as follows: rADG = (weight at d48 –weight at d26) / N * 1 / weight at d26, N being the exact number of days between these two visits. The rADG variable followed a normal distribution and was weakly correlated with ADG. Piglets were classified according to their rADG within their farm. In each farm, piglets showing the greatest rADG (top 40% of the distribution of 18 sampled piglets) and the lowest rADG (bottom 40% of the distribution) were assigned to the rADG+ and rADG- classes, respectively (approximately 7 piglets per class and per farm). Cutting out the middle 20% (3 to 4 individuals per farm) was a trade-off between the need to build contrasting groups in terms of growth aptitude (we hypothesized that the position of the 20% median piglets on either side of the median was likely to be random), without reducing too much the number of individuals available for the analysis.

### Bacterial DNA extraction and sequencing

A modified version of the protocol by Godon *et al*. [16] was used for DNA extraction. In summary, after a lysis step via incubation for one hour at 70°C with Guanidine Thiocyanate and N-lauryl sarcosine, bacterial DNA was extracted using the chemagic STAR DNA BTS Kit for the automated isolation of DNA (Perkin Elmer, Wellesley, MA, USA). Quality and purity of the isolated DNA were checked by spectrophotometry on the NanoDrop (Fisher Scientific, Schwerte, Germany). The V3-V4 hypervariable regions of the 16S rRNA gene were amplified with the primers PCR1F_460: CTTTCCCTACACGACGCTCTTCCGATCTACGGRAGGCAGCAG and PCR1R_460: GGAGTTCAGACGTGTGCTCTTCCG ATCTTACCAGGGTATCTAATCCT. The resulting PCR products were purified and sequenced using the Illumina MiSeq 2 x 300 bp kit (Illumina Inc., San Diego, CA, USA).

### Bioinformatics and biostatistics

The generated sequences were analyzed using a subsampled open-reference OTU strategy with default settings in QIIME (v1.9.1) [17]. The paired-end reads were merged and demultiplexed. Subsampled open-reference OTU-picking was carried out using UCLUST with 97% sequence similarity. The taxonomy of each representative sequence was assigned using the UCLUST method on the Greengenes database with a 90% confidence threshold. Data chimera were checked using the Blast fragments approach in QIIME [18]. Low quality samples with less than 5 000 reads were excluded, leaving 222 samples from d26 and 254 samples from d35, including 192 piglets having samples for both time points. The OTU table was rarefied and only OTUs of known phylum that were present in at least 5 fecal samples, and had a relative abundance greater than 0.01% of total reads of the 476 samples were considered. These samples contained information relative to 1 177 OTUs (minimum of 4 831 reads for d26, minimum of 4 958 reads for d35, and minimum of 4831 reads for the dataset merging d26 and d35 information, S2 Table).

The OTU table was imported in R computational language for the ecological parameters evaluation [19]. The variability within bacterial communities (alpha diversity) and the differences between bacterial communities (beta diversity) were calculated on the dataset composed of the merged data of d26 and d35, rarefied and filtered to 4 831 reads per sample using the “phyloseq” [20] and “Vegan” [21] packages. Both qualitative (observed richness) and quantitative (Shannon index) alpha diversity indexes were considered. The influence of the investigated factors (age, farm, and growth class) on body weight and alpha diversity indexes were analyzed by type 3 ANOVAs using the “lme4” [22] and “car” [23] packages on R software, using a model including the farm (16 farms), growth class, age (d26 and d35), and the interaction between growth class and age as fixed factors and the piglet as random factor. ADG and relative ADG were analyzed using a model that includes the farm (16 farms) and growth class as fixed factors.

For beta diversity, the Unifrac distance matrix was calculated. The effect of farm and age on the homogeneity of dispersion was determined using the betadisper function of Vegan. Then a permutation analysis of variance (Adonis procedure with 999 permutations) including age as a factor was carried out. The farm factor was excluded by the permutational analysis since the betadisper result was significant, meaning that the null hypothesis (farms have the same dispersions) can be rejected [24]. The distance matrix was visualized with non-metric multidimensional scaling (NMDS) plots.

Relative abundances at family and genus level were calculated on unrarefied data. Arumugam *et al*.’s methodology [25] was used to cluster samples from both time-points into groups of similar abundance called enterotypes. The clustering method was applied on the relative genus abundances. It used the Jensen-Shannon divergence distance and the Partitioning Around Medoids clustering algorithm. The optimal number of clusters was assessed using the Calinski-Harabasz Index. To evaluate the relevance of our enterotypes, the silhouette coefficient of our optimal clustering was compared to the silhouette coefficients obtained from 100 subsets generated by random sampling from our dataset.

The influence of the investigated factors (age, farm, and growth class) on relative abundances was investigated using nonparametric statistics. For the relative abundances of OTUs, the effects of age (d26 vs. d35) and rADG (rADG+ vs. rADG-), were tested on the logarithmic fold-change ratios of taxa using a Benjamini-Hochberg (BH) correction to control the false discovery rate, with the “edgeR” package of R [26]. For relative abundances aggregated at genus and family levels, the effects of factors with more than two levels (16 for the farm and 4 for the enterotype factor), were compared using Kruskal-Wallis and Dunn tests for each time point separately. The effect of rADG class was analyzed in two steps. In a first step pre-selected variables for which a rADG class effect seemed likely at least at one of the two time points. The rADG effect was tested separately at d26 and d35 using a Wilcoxon test with a BH correction, and a genus was kept for step 2 if the P-value was below 0.1 either at d26 or at d35. This threshold was chosen in order not to reduce too drastically the number of variables retained for the step 2. This pre-selection used all the data present for each time point in rADG classes–and + (178 observations with missing data for one farm on d26, and 208 observations with missing data for another farm on d35). This step “protected” the second step, which used only animals of rADG+ and–classes with data available at both time-points (151 pigs, from only 14 farms). The second step used a model including the interaction of rADG class with age, that was tested using the permutation tests of the lmPerm package of R software [27]. The effect of a factor was considered significant if P < 0.05.

### Prediction of rADG using microbiota

The best linear model calculating rADG between d26 and d48 from microbiota data was determined using the Leaps package on R software [28]. The procedure was applied to a dataset including relative abundances of all families and genera, and of a preselection of OTUs identified as predictors of the rADG class at d26 and d35 as variables, and all piglets having data for both time points (from 14 farms). These OTUs were identified using sparse Partial Least Square Discriminant Analysis with the MixOmics package [29]. To evaluate the robustness of the prediction, for each farm i, coefficients were calculated from the dataset of the 13 other farms, which were then used to predict the rADG of piglets from farm i, and the adjusted-R_i_^2^ was calculated. The R^2^ presented in the results section is the mean of the adjusted-R_i_^2^ of the 14 simulations corresponding to the 14 farms.

## Results

Farms are described in Table 1 and S1 Table. Feces samples were collected in 288 piglets raised in 16 different farms, just before and one week after weaning. For practical reasons, samples were missing from one farm before and from one other farm after weaning, and 14 farms had samples for both visits. After exclusion of low quality samples, the final dataset included data from 222 suckling (26-day-old) and 254 weaned (35-day-old) piglets, with a minimal number of 4 831 reads per sample after rarefaction and low abundancy filtering, and including 1177 OTUs (S2 Table). The OTUs belonged to 12 classes, distributed in 6 phyla, 21 orders (including 17 known orders), 43 families (including 33 known families), and 79 genera (including 45 known genera). *Firmicutes* and *Bacteroidetes* were the most abundant phyla at both ages and accounted for 63% and 29% of total sequences for both ages together, followed by *Proteobacteria* (5.2%), *Spirochaetes* (1.7%), and *Fusobacteria* (1.3%).

### Differences between pre- and post-weaning microbiota

**Fig 1:**
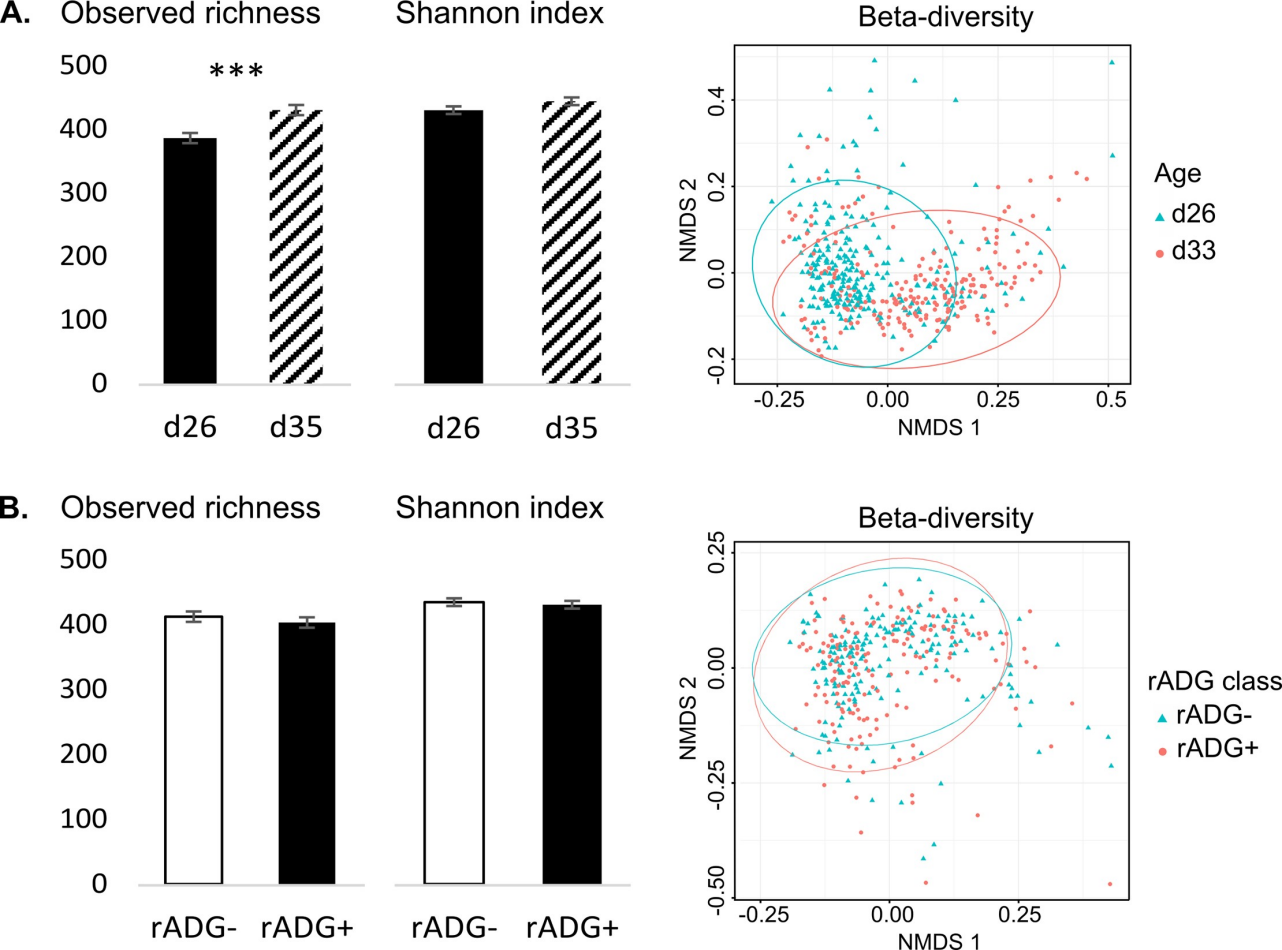
Influence of age (A) and rADG class (B) on alpha and beta diversity of piglet fecal microflora. Samples were collected from suckling 26-day-old (n = 222) and weaned 35-day-old piglets (n = 254) from 16 commercial farms. They were classified according to their relative ADG (rADG) between d26 and d48 into high (rADG+) or low (rADG-) growth classes and the 20% pigs showing a median rADG were excluded. Alpha diversity was described by observed richness and Shannon index and beta diversity was expressed according to the non-metric multidimensional scaling (NMDS) on Unifrac distances calculated at the OTU level. For observed richness and Shannon index a significant effect of age or rADG class is indicated by * (P <0.001).

Both the observed richness and Shannon diversity index increased slightly 7 days after weaning (P < 0.01, Fig 1 and Table 2). As for beta diversity, a variation in the composition of bacterial communities was observed by piglet age (Adonis test: P < 0.001), and samples partially clustered by age, as shown in the NMDS plot (Fig 1).

**Table 2:** Influence of age, relative average daily gain, and microbial enterotype on alpha diversity indexes of fecal microbiota.

	Observed richness			Shannon index		
Factor	Factor level	Mean (SEM = 8)	Factor P-value ^3^	Factor level	Mean (SEM = 0.06)	Factor P-value ^3^
*Age*^*1*^	D26	386^a^	<0.001	D26	4.30 ^a^	0.035
	D35	430^b^		D35	4.44 ^b^	
*rADG class*^*2*^	rADG-	412	0.460	rADG-	4.39	0.533
	rADG+	403		rADG+	4.35	
*Farm*			<0.001			0.002
*rADG x Age*			0.218			0.171
*Enterotype*	E1	264^a^	<0.001	E1	3.75^a^	<0.001
	E2	372^b^		E2	4.29^b^	
	E3	507^c^		E3	4.92^c^	
	E4	422^b^		E4	4.40^b^	

^1^Samples were collected from suckling 26-day-old (n = 222) and weaned 35-day-old piglets (n = 254) from 16 commercial farms.

^2^In each farm, animals belonging to the 40% lowest and 40% highest percentiles of relative average daily gain (rADG), as calculated between d26 and d48, were assigned to rADG- or rADG+ classes.

^3^ANOVAs including the effect of the factor, age, and their interaction were performed. There was no significant interaction between age and the tested factors.

**Fig 2:**
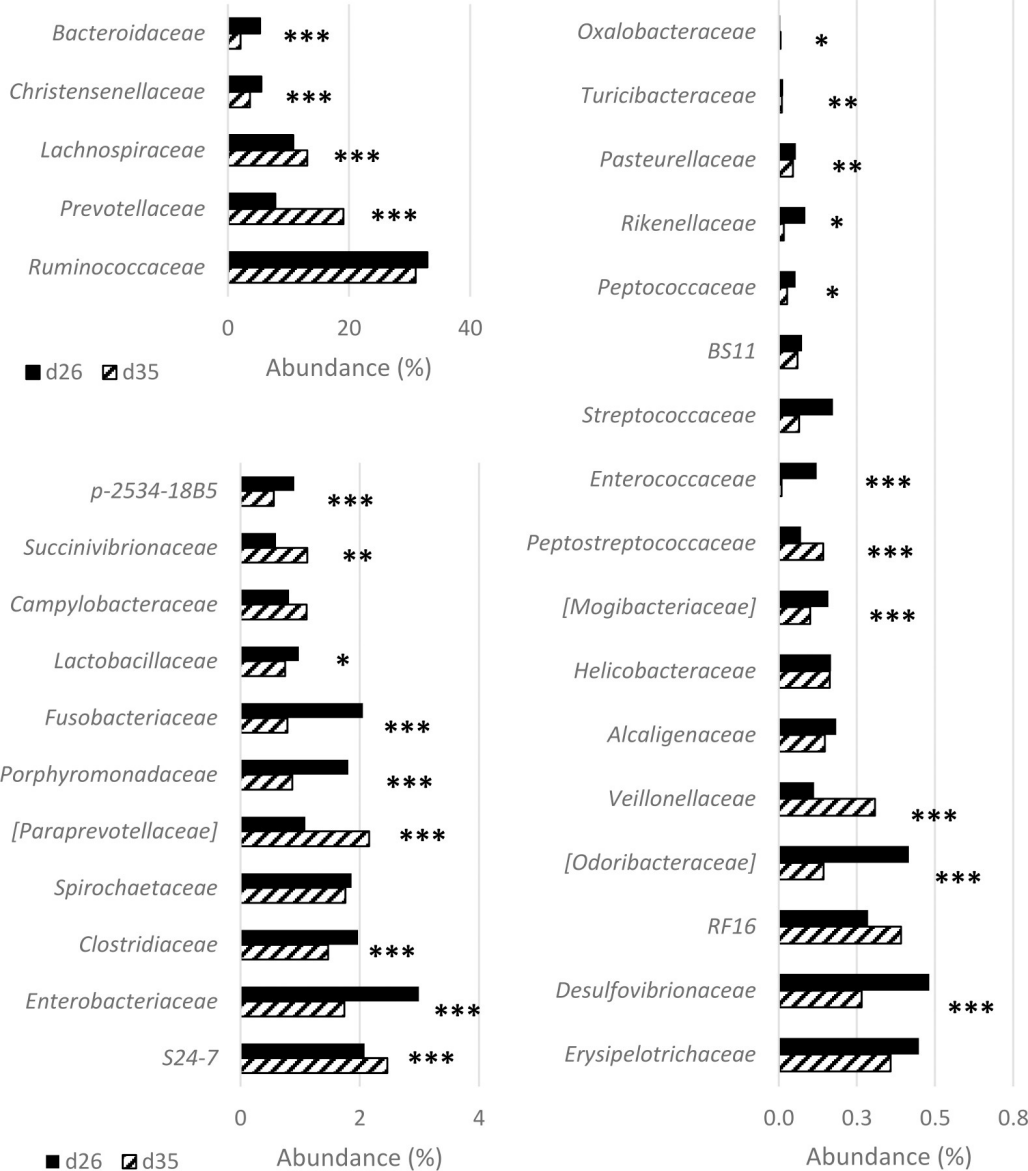
Relative abundances of the 33 families detected in piglet fecal samples before and after weaning. Samples were collected from suckling 26-day-old (n = 222) and weaned 35-day-old piglets (n = 254) from 16 commercial farms. For each taxon a significant effect of age is indicated by ***(P <0.001) ** (P <0.01) or *(P <0.05).

The comparison of the relative abundance of taxa indicated a clear effect of age, with 950 of the 1177 OTUs having a fold change significantly affected by age (FDR < 0.05, 514 decreased and 436 increased after weaning). These OTUs belonged to the 33 represented families (S3 Table and S1 Fig). The relative abundances of 16 of these families decreased after weaning and the relative abundance of 8 families increased (P < 0.05, Fig 2). *Bacteroidaceae* (mainly genus *Bacteroides*) decreased by 61% after weaning (P < 0.001), including the species *B*. *fragilis*, *B*. *plebeius*, and *B*. *uniformis*. *Christensenellaceae* (-35%, *P < 0*.*001)*, *Enterobacteriaceae* (- 42%, P < 0.001) and *Clostridiaceae* (-32%, P < 0.001) decreased after weaning (Fig 2). *Prevotellaceae (*mainly *Prevotella)* increased by +143% (P < 0.001). The relative abundance of *Lachnospiraceae* generally increased (+21%, P < 0.001), especially the genera *Blautia* (+69%), *Lachnospira* (+125%) and *Roseburia* (+150%, P < 0.001). The relative abundance of *Ruminococcaceae* was stable over time, but in this family, *Faecalibacterium* increased after weaning (+117%, P < 0.001, and *F*. *Prausnitzii* in particular), *Oscillospira* decreased (-14%, P < 0.05), and *Ruminococcus* decreased (-22%, P < 0.05, especially *R*. *Gnavus)*. Some other less abundant families such as *Porphyromonodaceae* (-52%), *Fusobacteriaceae* (-62%), *Enteroccaceae* (-93%), *Rikenellaceae* (-81%), *Succinivibrionaceae* (+87%), *Veillonellaceae* (+173%), and *Peptostreptococcaceae* (+100%) displayed large variations (P < 0.05) in relative abundance on d35 compared with d26.

### Determination of enterotypes

**Fig 3:**
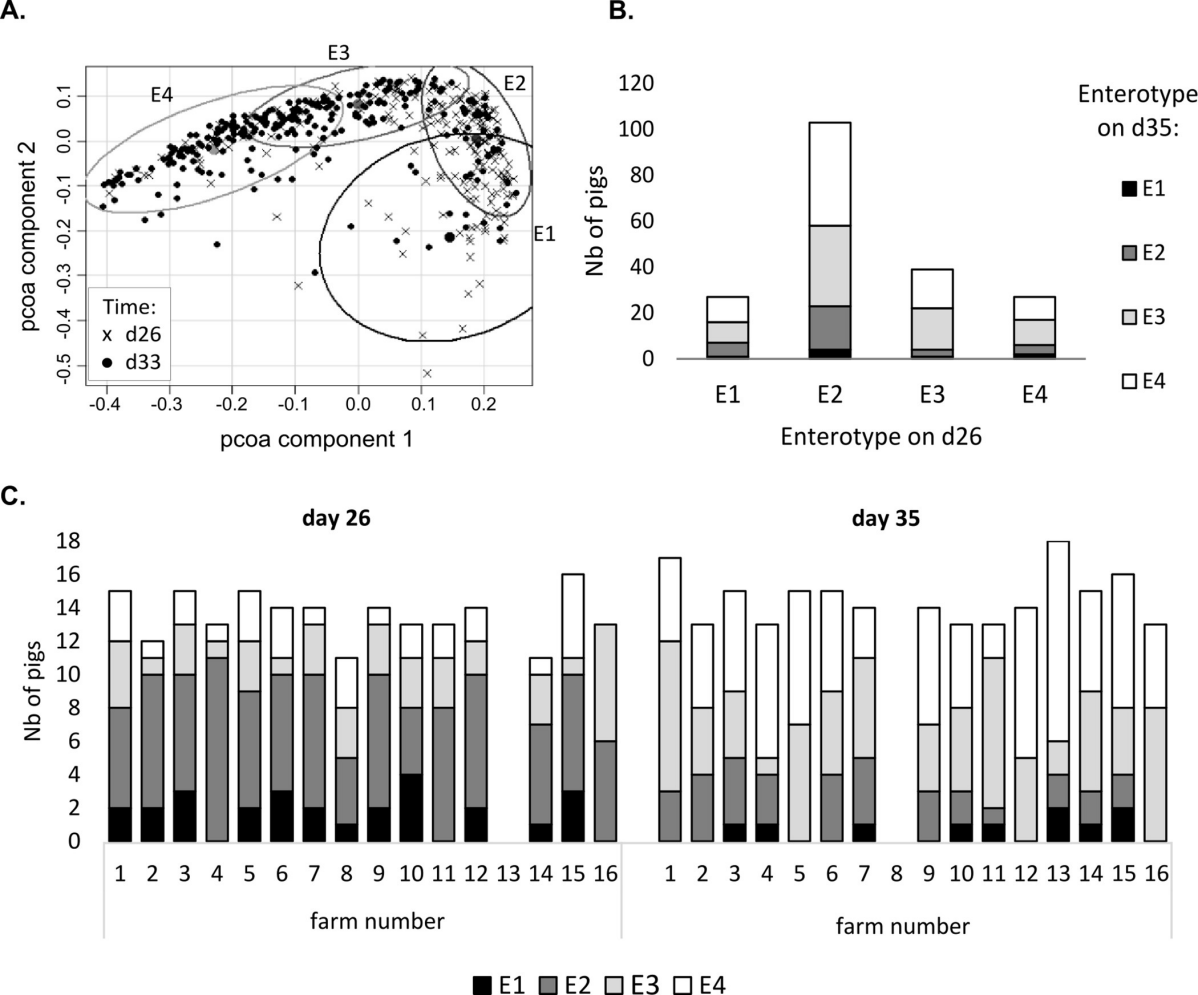
Enterotypes identified in fecal samples from piglets. Four enterotypes named E1 to E4 (A) were identified among the samples collected from suckling 26-day-old (n = 222) and weaned 35-day-old piglets (n = 254) in 16 commercial farms. The 192 pigs with data available at both time points were used to show the proportion of pigs switching from one enterotype to another between d26 and d35 (B). The variability between farms is illustrated by the distribution of enterotypes at d26 and d35 in each farm (C).

Four enterotypes (E1 to E4) were established (Fig 3A), for which the most discriminating genera were *Prevotella*, *Faecalibacterium*, *Roseburia*, and *Lachnospira* (Table 3). Both E1 and E2 showed lower relative abundances of these four genera compared with E3 and E4. E4 had the highest relative abundances of these four genera. Based on the number of OTUs and the Shannon index, diversity increased between the enterotypes from E1 to E3 (P < 0.001, Table 2). Between d26 and d35, 75% of the piglets shifted from one enterotype to another (Fig 3B). This dynamic shift evolved from E1 to E2, then to E3, and finally to E4.

**Table 3:** Relative abundance of genera discriminating the E1, E2, E3, and E4 enterotypes.

	Mean relative abundance (%)				Enterotype
Genus^1^	E1	E2	E3	E4	effect (P-value)^2^
*Prevotella*	3.87a	1.32a	11.29b	31.4c	<0.001
*Faecalibacterium*	0.35a	0.24a	2.41b	11.08c	<0.001
*Roseburia*	0.24a	0.17a	1.20b	4.36c	<0.001
*Lachnospira*	0.02a	0.05a	0.48b	1.08c	<0.001
*Bacteroides*	16.10a	5.71b	0.59c	0.63d	<0.001
*Coprococcus*	0.12a	0.35b	0.86c	0.93c	<0.001
*Treponema*	0.54a	1.21b	3.77c	1.09b	<0.001
*Paludibacter*	0.02a	0.26b	0.27c	0.06a	<0.001
*Ruminococcus*	1.27a	2.25b	2.16c	1.34a	<0.001
*Mitsuokella*	0.02ab	0.01a	0.03b	0.21c	<0.001
*Oscillospira*	2.43a	3.59b	2.79c	2.05a	<0.001
*Lactobacillus*	1.82a	0.88bc	0.78c	0.68d	<0.001
*Campylobacter*	1.52a	0.57b	1.14a	1.11a	<0.001

^1^The table presents a non-exhaustive list of genera that were differently expressed among enterotypes. Means were calculated using the total dataset from suckling 26-day-old (n = 222) and weaned 35-day-old piglets (n = 254) raised in 16 commercial farms.

^2^The order of genera in the table follows the P-value of the Kruskal-Wallis test for the enterotype effect (greatest for *Prevotella*, lowest for *Campylobacter*).

### Influence of farm environment on piglet microbiota

**Fig 4:**
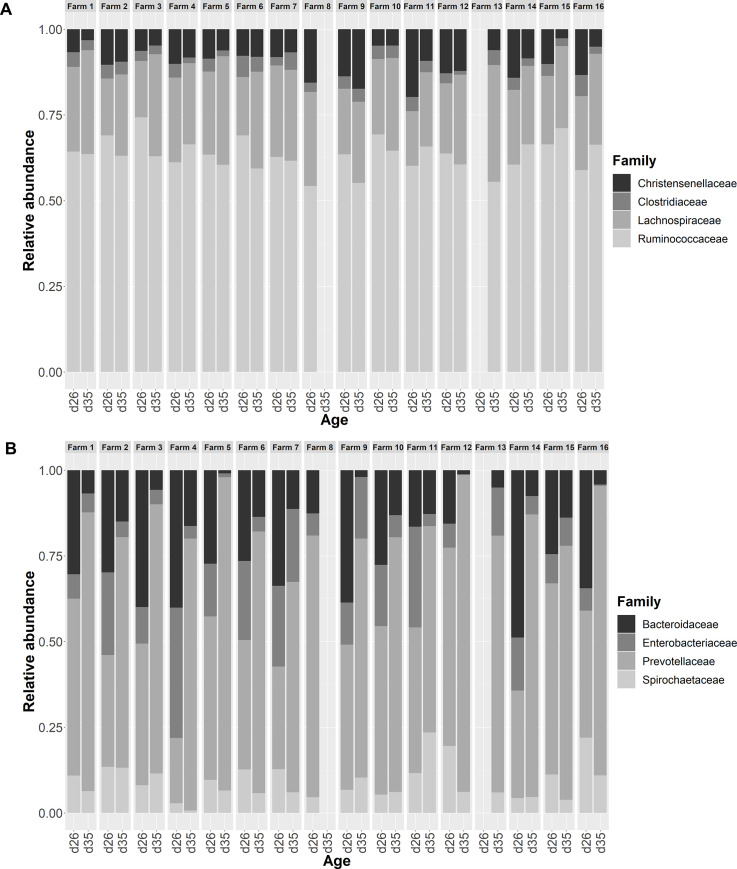
Variability between farms of the relative abundancies of the first 8 most abundant families in piglet fecal samples. Samples were collected from suckling 26-day-old (n = 222) and weaned 35-day-old piglets (n = 254) from 16 commercial farms. Families from *Firmicutes* (A) as well as *Bacteriodetes* and *Spirochaetes* phyla (B) are presented. Among these families before weaning farm influenced only the relative abundance of *Christensenellaceae* (P < 0.05). After weaning abundancies of *Ruminococcaceae Lachnospiraceae Clostridiaceae Entrobacteriaceae* and *Spirochaetaceae* were also different among farms (P < 0.05).

At both d26 and d35, richness varied among farms but not Shannon index diversity (P < 0.05 and > 0.1, respectively). The comparison of the relative abundances of microbial families among farms revealed statistically significant differences, indicating a variability associated with the farm. Before weaning (d26), farms differed in the relative abundance of *Christensenellaceae*, which was the 5^th^ most abundant family in piglet microbiota, and for 5 other less abundant families, including *Lactobacillaceae* (P < 0.05, S4 Table). After weaning (d35), the differences due to the environment increased, since a farm effect was significant for the relative abundances of 21 families (P < 0.05). The 4 enterotypes were observed in all farms, at least at one of the two different ages (Fig 3C), but the distribution of enterotypes was heterogeneous among farms at d35 (P < 0.05). Despite these differences in relative abundances, the effect of weaning on the most abundant microbial families was roughly the same in all farms (Fig 4). For example, the weaning-associated decrease in the relative abundances of *Bacteroidaceae*, *Christencellaceae*, *Enterobacteriaceae*, and *Clostrididiaceae* was numerically observable in 14, 12, 13, and 12 farms respectively, of the 14 having data for both time points, and the increase in the relative abundances of *Prevotellaceae* and *Lachnospiraceae* was numerically observable in 14 and 12 farms, respectively (S4 Table).

### Classes of relative average daily gain (rADG)

Because individual daily health recordings are difficult to obtain in field studies using large numbers of animals, the growth performance of the piglets was used as a proxy for their robustness. The growth performance of the pigs from the 16 farms is shown in Table 1. Piglets are fast growing animals whose ADG over a given period of time is highly correlated to the body weight at the beginning of the study period. We believe that ADG is a bad indicator of individual robustness to a specific event (here, weaning), because it is so strongly correlated to the starting weight that the effects of other factors are overwhelmed. To obtain a growth indicator that only reflects the adaptive capacity of piglets to weaning and not the influence of pre-weaning growth, we used the relative ADG (the ratio between ADG over the d26 to d48 period and weight at d26, rADG). In each farm, piglets showing the greatest rADG (top 40% of the 18 sampled piglets) and the lowest rADG (bottom 40% of the 18 sampled piglets) were assigned to the rADG+ and rADG- classes. Among them, 106 rADG+ and 106 rADG- pigs had microbial data at least at one time point, and 72 rADG+ and 76 rADG- pigs had microbiota data for both time points.

Relative ADG was different in the two rADG classes by design (rADG+: 47.2 vs. rADG-: 28.7 ± 1.7 g/kg/day, P < 0.001). The ADG was lower in rADG- compared to rADG+ pigs, (rADG+: 0.367 vs. rADG-: 0.247 ± 0.018 kg/day, P < 0.001) but the weight at weaning was greater in rADG- pigs (rADG+: 7.79 vs. rADG-: 8.53 ± 0.23 kg, P < 0.001).

### Relationship between piglet growth after weaning and microbial profile

**Fig 5:**
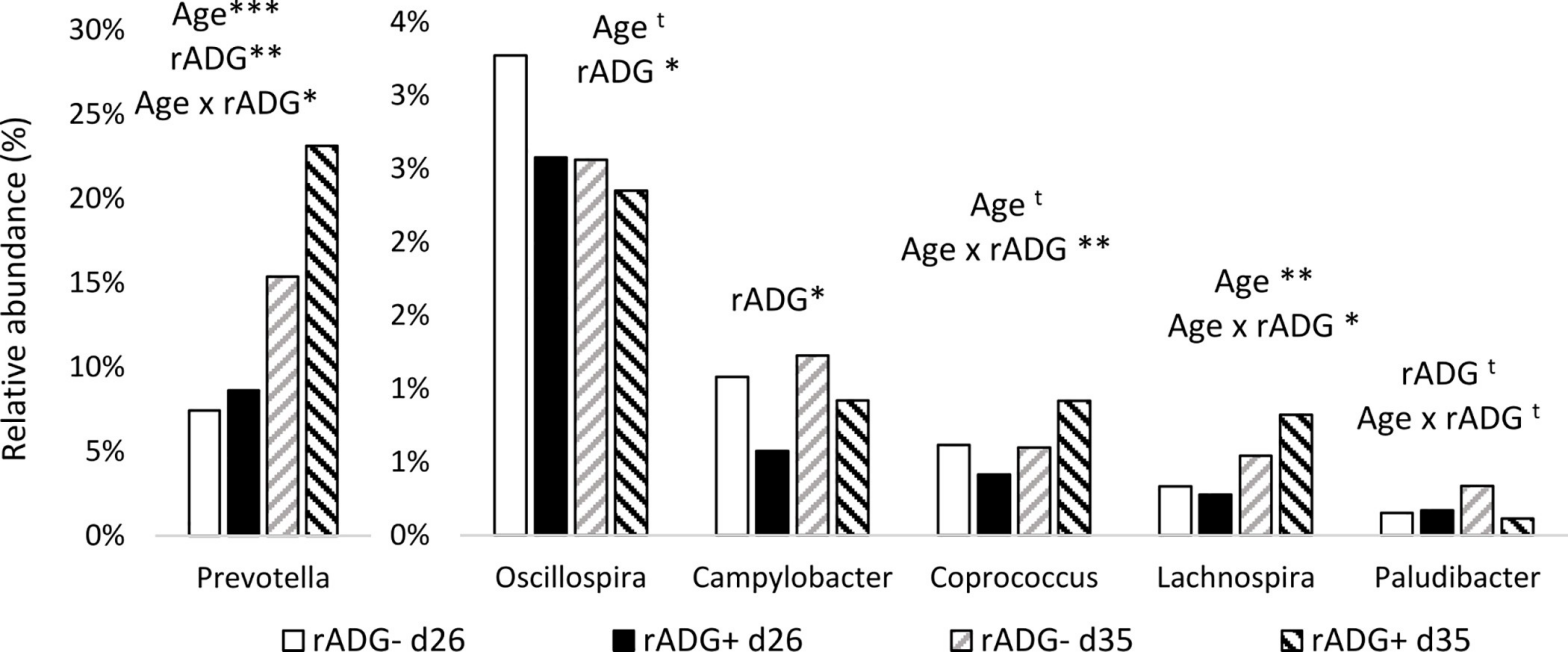
Relative abundances of significant genera and families between pigs with high or low post-weaning growth. Fecal samples were collected from piglets at 26 (222) and 35 days of age (254). Piglets were classified according to their relative ADG (rADG) between d26 and d48 into high (rADG+) or low (rADG-) growth classes and the 20% pigs showing a median rADG were excluded. For each taxon trends and significant effects of age rADG class and their interaction are indicated. ***(P < 0.001) ** (P < 0.01) *(P < 0.05) and t (P < 0.1).

The Shannon index and the observed richness were similar in rADG- and rADG+ classes, and beta diversity (Adonis test: P > 0.1) indicated a close composition between bacterial communities of both classes (Fig 1 and Table 2). Nevertheless, pigs in the rADG+ group differed from pigs in the rADG- group for 315 OTUs at d26 (P < 0.05, 150 increased and 165 decreased in rADG+), and for 461 OTUs at d35 (P < 0.05, 244 were increased and 217 decreased in rADG+), which were distributed among 14 and 15 different families, respectively (S5 Table). At phylum level, whatever the age, rADG+ pigs had more *Bacteroidetes* (31.5 vs. 27.2%, P < 0.01) and less *Proteobacteria* (4.18 vs. 6.65%, P < 0.001) than rADG- pigs. Differences were also detectable at the genus level: rADG+ pigs had less *Oscillospira* (P < 0.05) and *Campylobacter* (P < 0.01) than rADG- pigs (Fig 5). There was a time x rADG group interaction for *Prevotella* (P < 0.01), *Coprococcus* (P < 0.01), and *Lachnospira* (P < 0.05), whose relative abundances increased after weaning, and for which the increase was even more visible in rADG+ pigs. The relative abundance of *Paludibacter* tended to be influenced by the time x rADG group interaction (P < 0., Fig 5). As for enterotypes, compared to piglets that had reached the E4 enterotype 7 days after weaning, piglets at the E2 enterotype and E3 enterotypes had or tended to have a lower rADG (E4: 43.5 ± 1 vs. E2: 37 ± 2 g/kg/day, P < 0.05 and vs. E3: 40 ± 1 g/kg/day, P < 0.1). E1 pigs had an intermediate rADG (42 ± 3 g/kg/day).

The absolute value of rADG between d26 and d48 could be predicted by a linear model using data from microbiota at d26 and d35 with an adjusted R^2^ of 0.29 (Table 4). The model included the relative abundances of the *Porphyromonodaceae* family at d26 and d35 (P < 0.001 and < 0.05), the relative abundance of three genera (*Campylobacter* at d26, P < 0.01; *Fusobacterium*, P < 0.001; *Clostridium* at d35, P < 0.05), and of 4 OTUs belonging to *Ruminococcaceae* (OTU 4315785 at d26, P < 0.05), *Coprococcus* (OTUs 295683 and 752354 at d35, P < 0.001 and 0.05), and *Prevotella* (OTU 350627 *Prevotella copri* at d35, P < 0.001). The robustness of this prediction was bolstered by cross-validation among the 14 farms having data for both time points (see the biostatistics section of the material and methods). The obtained R^2^ remained moderate, but this prediction was nevertheless much better than the one obtained from weight at d26 alone, which had R^2^ of 0.03, showing that body weight before weaning is not a predictor of relative growth (robustness) after weaning.

**Table 4:** Estimates of the parameters of the linear model calculating rADG over the d26-d48 period.

Variable	Taxonomic classification	Estimate^1^	Std. error^1^	P-value
(Intercept)		0.038	0.001	<0.001
*Campylobacteraceae*, d26	f. *Campylobacteraceae*	-0.001	0.000	<0.01
*Porphyromonadaceae*, d26	f. *Porphyromonodaceae*	0.001	0.000	<0.001
*Porphyromonadaceae*, d35	f. *Porphyromonodaceae*	-0.001	0.000	<0.05
*Clostridium*, d35	f. *Clostridiaceae*, g. *Clostridium*	-0.017	0.008	<0.05
*Fusobacterium*, d35	f. *Fusobacteriaceae*, g. *Fusobacterium*	0.001	0.000	<0.001
OTU_4315785, d26	f. *Ruminococcaceae*, g. unknown	-0.029	0.011	<0.05
OTU_295683, d26	f. *Lachnospiraceae*, g. unknown	-0.085	0.021	<0.001
OTU_752354, d26	f. *Lachnospiraceae*, g. *Coporococcu*s	0.005	0.002	<0.05
OTU_350627, d26	f. *Prevotellaceae*, g. *Prevotella*, s. *Copri*	0.01	0.003	<0.001

^**1**^The dataset used to build the equation encompassed data from the 192 pigs for which data were available at both time points (pre-weaning at day 26, and post-weaning at d35). The model included the relative abundances of a few taxonomic groups at d26 and d35 and predicted rADG with an adjusted R^2^ of 0.29.

## Discussion

### Microbial maturation and enterotypes around weaning

Our study was distinctive in that it investigated the evolution of piglet microbiome after weaning in 16 different commercial farms, including data from 222 male piglets before and 254 piglets after weaning, respectively. These different environments made it possible to test the universality of the changes induced by weaning transition previously reported in the literature [7, 30, 31]. The alpha diversity values increased post-weaning compared to pre-weaning, which is recognized as an indicator of greater stability and microbial maturity [30, 32]. A decrease in the relative abundances of *Enterobacteriaceae*, *Bacteroidaceae*, *Clostridiaceae*, *Enterococcaceae*, *Fusobacteriaceae*, *Porphyromonodaceae*, *and Rikenellaceae* families and *Bacteroides*, *Fusobacterium*, *Ruminococcus* genera, as well as an increase in *Prevotella*, *Veillonellaceae*, and *Succinovibrionaceae* was observed after weaning. It is notable that this recognized microbial shift [33] was observed despite the fact that pre and post-weaning time points were close (d26 and d35), indicating a rapid shift in the bacterial community.

Mach *et al*. [7] found that maturing bacterial communities in piglets evolved after weaning toward two different clusters, primarily distinguished by their relative abundances in unclassified *Ruminococcaceae* and *Prevotella*. Pre-weaned pigs all belonged to the *Ruminococcaceae* cluster and the shift toward the *Prevotella* cluster started at weaning, but the two phenotypes persisted in older fattening pigs [34]. Using the same pipeline, based on the methodology developed by Arumugam *et al. [25]*, we identified four enterotype-like clusters in our set of samples collected around weaning that appeared to be ubiquitous since they were observed across the all 16 farms. The shift from E1 to E4 was associated with an increase in the relative abundance of *Faecalibaterium*, *Lachnospira*, *Prevotella*, and *Roseburia* genera. Piglets were distributed in all enterotypes before weaning, and a majority of them shifted after weaning to enterotype 3 or 4. Thus, the transition from E1 to E4 probably reflected the stages of increasing microbiota maturity from the suckling phase to a more mature microbiota that can use the new nutritive substrates provided by a cereal-based diet. According to their relative abundances of *Prevotella*, *Ruminococcus*, *Mitsuokella*, and *Treponema*, the four enterotype-like clusters observed in the present study could all belong to the “*Ruminococcus* and *Treponem*a” enterotype described previously [34]. This is consistent with the age of the pigs and the very short time elapsed since weaning in the present study. Indeed, the shift toward the “*Prevotella*” phenotype develops slowly after weaning [7].

### Inter-farm variability of the microbial profile of piglets

In the present study, 16 commercial farms were included in order to determine the extent to which variations in farm environment and rearing conditions influence the structure of piglet microbiota and its evolution after weaning. The variability of the farms was controlled since all the selected farms were indoors, geographically located in the West of France, weaned their piglets at 4 weeks of age, and used the same breed. These farms were representative of the “dominant model” of commercial farms in this region, and covered the inter-farm variability in this dominant system regarding feeding practices, pen size and design, control of thermic environment, antimicrobial practices, and pathogen presence in the herd (see S1 Table). As stressed by others [14], an effect of farm on microbial profile was expected because these factors may influence the gut bacterial community of piglets. Indeed, in addition to animal age and genetic [35–37], the structure and activity of gut microbiota can differ between animals depending on various other factors including diet [5, 38], status regarding specific pathogens [39], antimicrobial use [32, 40–42], and season of the year [43]. Physical and hygienic characteristics of the housing environment also play an important role in shaping the gut microbiota. This has been demonstrated by comparing the gut microbiota of piglets raised indoors vs. outdoors [44], mice raised in a clean environment vs. in the presence of dust and soil [45], and human babies exposed to different house dusts [46]. The experiment was not designed to individually explore the influence of each of these factors, which were too numerous to be statistically analyzed independently with data from only 16 farms. Among suckling piglets, farms differed in the relative abundances of *Christensenellaceae* and *Lactobacillu*s, which are common genera in the pig hindgut and are considered to be beneficial bacteria that can improve gut health or feed efficiency [4, 47]. After weaning, farms differed from one another in the relative abundances of two thirds of their present microbial families, indicating that the living environment has more influence on the structure of fecal microbiota after weaning than before. However, despite differences, the effect of weaning on the most abundant microbial families was roughly the same in all farms: decreased relative abundances of *Bacteroidaceae*, *Christencellaceae*, *Enterobacteriaceae*, and *Clostrididiaceae*, and increased relative abundances of *Prevotellaceae* and *Lachnospiraceae*.

### Association between relative growth and microbiota

Robustness is the capacity to maintain productivity in a wide range of environments without compromising reproduction, health, and welfare [48]. It is not easy to record individual clinical signs of disease in herds of pigs on a daily basis. In the present study, farmers only reported the presence of clinical signs at the level of the room where animals were housed. However, in piglets, which are young and fast growing animals, analyzing the growth trajectory after weaning can easily reveal health disturbances [3, 49]. Thus, we used the post-weaning growth performance (ADG) corrected by weaning weight. We assumed that the relative ADG (rADG) would be a better proxy for the adaptation after weaning than ADG, because it corrects from the influence of weight at weaning on subsequent mass accretion.

We developed two approaches to identify microbial signatures that would be predictive of rADG. One was based on the construction of linear models aiming at predicting rADG from the relative abundances of specific OTUs, genera or families, without including any information related to the farm from which each pig originated. The absolute value of rADG between d26 and d48 could be predicted with an adjusted R^2^ of 0.29. The growth of a young animal is influenced by various factors that are related to processes occurring in the gut (feed composition, intake and digestibility, gut health) or not (global health of the animal, genetic potential, energy expenditure for thermoregulation and physical activity, etc). The present result indicates that nearly one third of this inter-individual variability could be predicted by two measures performed in feces around weaning, and highlights the strong relationship between the structure of the digestive microbiota and the biological processes controlling piglet’s growth. However, this predictive model included the relative abundance of taxonomic groups that were highly influenced by the farm (*Porphyromonodaceae*, *Campylobacteraceae*, *Clostridiaceae*). Since rADG was also significantly influenced by the farm, these microbial differences might be more farm signatures than signatures of animal growth abilities.

Thus, a second approach was developed to identify common microbial signatures characterizing robust piglets. In each farm, animals showing low and high rADG relative to their pen-mates were assigned to low and high rADG classes. This classification was used to look for characteristics of the microbiota that would be shared by robust piglets independently of their rearing environment, including the use (or not) of antimicrobials. We observed that, regardless of age, the animals having the highest rADG in their farm had a higher relative abundance of *Bacteroidetes* and a lower relative abundance of *Proteobacteria* than their pen-mates who grew more slowly. Since increased abundance of *Proteobacteria* is considered to be a potential indicator of gut dysbiosis [50, 51], the most robust piglets may have a microbiota that is more resistant to dysbiosis, perhaps thanks to commensal bacteria that competitively exclude pathogens. At genus level, they had a higher relative abundance of *Prevotella* post-weaning, which has been reported to be positively correlated with ADG [7, 8]. This greater abundance in better growing animals could be related to the capacity of *Prevotella* to produce enzymes that degrade complex dietary polysaccharides, thus improving fiber digestibility and host feed efficiency [4, 5]. The abundance of *Prevotella* has also been associated with resistance to PWD [11]. Finally, piglets of the rADG+ class also had more *Coprococcus* and *Lachnospira*, and less *Oscillospira* and *Campylobacter* compared to their farm-mates. The relationship between these genera and robustness of animals has not been reported before. However, both *Coprococcus* and *Lachnospira* belong to the order of *Clostridiales* and are known for the short chain fatty acid they produce, and specifically, for their butyrate-producing ability that can promote the differentiation of regulatory T cells and has an anti-inflammatory function [52, 53]. In a recent study, the abundance of *Oscillospira* in post-weaning piglets predicted the excretion of *Salmonella*, but the reason why was not determined by authors. They proposed that a greater abundance of *Oscillospira* could be an indicator of immaturity of the microbiota [54]. Interestingly, the rADG+ phenotype shared characteristics with the E4 enterotype (more *Prevotella*, *Coprococcus*, and *Lachnospira*). In the same line, animals displaying the E4 enterotypes 7 days after weaning indeed had a higher relative growth than animals in the E2 and E3 enterotypes. This suggests that a faster maturation of microbiota after weaning would be associated with better robustness. The fact that this microbial signature was found in individuals from several different farms is a guarantee of the robustness of this observation. However, the study was carried on in male piglets, and extrapolating our results to females should be done with caution, as gender can influence the fecal microbiome of pigs [55]. Furthermore, these results only indicate associations and do not prove any causal relationships.

## Conclusion

In this study, the effect of weaning on fecal microbiota was confirmed in a variety of commercial farms homogenous regarding their geographical localization, breed and weaning age, but differing in the distributed feeds, management practices, and pathogenic status. We observed the presence of ubiquitous enterotypes among farms, reflecting maturational stages of microbiota from a young suckling to an older cereal-eating profile. This maturation may be associated with the robustness of piglets. Indeed, regardless of the farm where they were raised, piglets whose growth was less disrupted by weaning were those who had reached the more mature phenotype characterized by *Prevotella* the fastest.

## Supporting information

S1 Fig Influence of age on the representation of microbial families. The figure presents, in each family, the proportions of OTUs for which the relative abundance at d35 significantly decreased (LogFC < 0 P < 0.05, n = 514) or increased (LogFC > 0 P < 0.05, n = 436) compared to d26. Fecal samples were collected from suckling 26-day-old (n = 222) and weaned 35-day-old piglets (n = 254) from 16 commercial farms. The numbers in brackets indicate the total number of OTUs present in each family.
(JPG)

S1 Table Detailed characterization of the farms practices and treatments during the study and growth of animals involved in the study. (XLSX)

S2 Table Description of the dataset. The numbers and characteristics (minimal number of reads and number of OTU) of the microbiota dataset are presented. Datasets after rarefaction were used to study alpha diversity. Datasets not rarefied but filtered were used to do the other analyses.
(XLSX)

S3 Table Influence of age on the number of differentially expressed OTUs per family and on the relative abundance of each family in piglet fecal samples. (XLSX)

S4 Table Influence of farm on the relative abundance of each family in fecal samples of piglets. (XLSX)

S5 Table Influence of rADG class on the relative abundance of each family in fecal samples of piglets. (XLSX)
