# Supplementary figures and images for: The fecal microbiota of piglets during weaning transition and its association with piglet growth across various farm environments
Source: PLoS One. 2021 Apr 27;16(4):e0250655. doi: 10.1371/journal.pone.0250655 (PMC8078812; doi:10.1371/journal.pone.0250655)

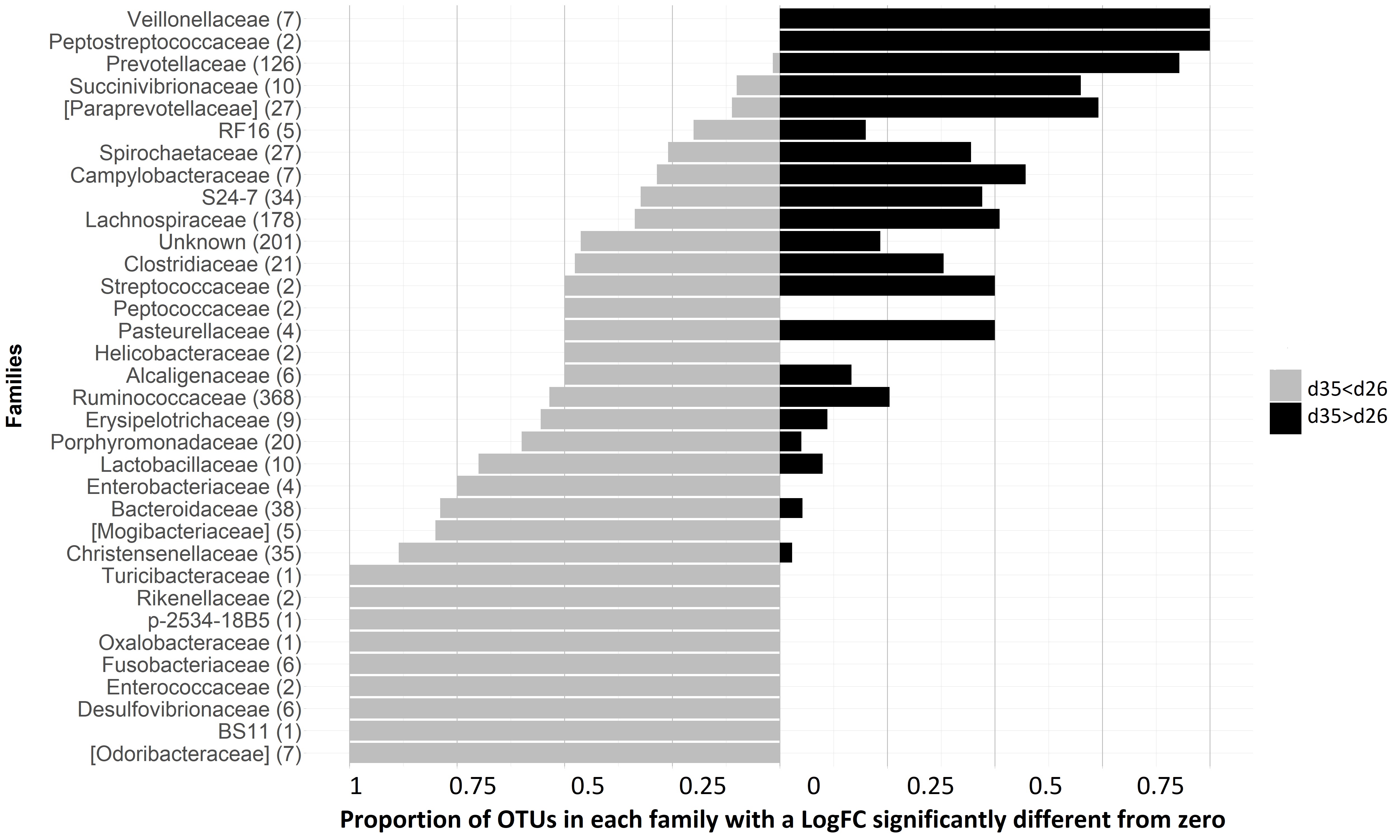

Supplement: S1 Fig — The figure presents, in each family, the proportions of OTUs for which the relative abundance at d35 significantly decreased (LogFC < 0 P < 0.05, n = 514) or increased (LogFC > 0 P < 0.05, n = 436) compared to d26. Fecal samples were collected from suckling 26-day-old (n = 222) and weaned 35-day-old piglets (n = 254) from 16 commercial farms. The numbers in brackets indicate the total number of OTUs present in each family. (JPG) [file pone.0250655.s001.jpg]
